# Curious Case of Ascending Paralysis

**Published:** 1830-01-01

**Authors:** 


CURIOUS CASE OF ASCENDING PARALYSIS; WITH THE APPEARANCES ON DISSECTION.

*Case.* Charles L. 35 years of age, robust, and in the military profession for 14 years, during which he had served in the Russian and Spanish campaigns, and consequently been exposed to great fatigues and vicissitudes of climate. In June, 1826, he first perceived that his legs readily bent under him, and that he could not easily raise himself up from the sitting posture. In other respects he had no complaint. In about a fortnight after this he began to feel numbness in his feet, which gradually ascended towards the knee. But while the surface thus lost its sensibility, the muscles beneath became the seat of acute pain, which was much exasperated by pressure. He had been a month confined to bed in this state, with nearly loss of all power in the lower extremities, when he perceived a numbness invade his hands. The progress was exactly similar to that in the inferior members; and he was seen by the narrator on the 22d of September of the same year. He was now completely paralytic, excepting the tongue, the face, and the neck. These last became gradually affected. He had never complained of pain in his head, nor of any part of the spine; nor did the most rigid examination detect any physical lesion in this last region. His general health was good—his intellects perfect. He attributed his disease to rheumatism, contracted during his bivouacs in Spain. He made water voluntarily, and had a daily evacuation from the bowels. He slept and ate well. The skin was nearly of natural temperature, but quite insensible to pinching or pricking. Any pressure of the muscles, on the other hand, gave him great pain, and caused him to cry out.

Frictions of lytta and alcohol were assiduously employed along the spine—ammoniated liniments were applied to the limbs, and cinchona, with wine, was liberally exhibited internally. In the course of a fortnight the sensibility of the skin began to return and that of the muscles to diminish. The power of the muscles also gradually returned, but inversely to the way in which it had been lost—namely, from above downwards. He was never able, however, to raise himself up on his feet. This amelioration continued but for a very short time, and he was soon as bad as ever. Blisters along the spine were added to the former measures. On the 3d of November he became suddenly incommoded in his breathing—his pulse quickened—his countenance became anxious—he had cough—the intercostal muscles seemed scarcely to move. In this state he lingered till the 7th of the same month, when he expired without any struggle.

*Dissection.* The spinal canal was opened throughout its whole extent. There was very little blood in the venous sinuses. The dura mater in its natural state. The pia mater was sprinkled with calcareous depositions in the lumbar region, and was finely injected. The roots of the lumbar and sacral nerves, as also the great sciatic, were injected with black blood. The other nerves were very minutely examined; but nothing particular was observed. The spinal marrow was rather firmer than natural, and the same might be said of the medulla oblongata and brain. The lungs were filled with tuberculous matters, and there were some small abscesses. The heart was empty and flaccid. The whole of the abdominal viscera were sound. The muscles presented no appearance different from those of a person in health, except being more pale and flaccid.

The foregoing case will shew with what a thick veil the functions and diseases of the nervous system are veiled. What was the nature of the malady? Was it inflammatory—or was it the reverse?—Clinique.

---

